# Biological Control of Potato Common Scab With Rare Isatropolone C Compound Produced by Plant Growth Promoting *Streptomyces* A1RT

**DOI:** 10.3389/fmicb.2018.01126

**Published:** 2018-05-30

**Authors:** Arslan Sarwar, Zakia Latif, Songya Zhang, Jing Zhu, David L. Zechel, Andreas Bechthold

**Affiliations:** ^1^Department of Microbiology and Molecular Genetics, University of the Punjab, Lahore, Pakistan; ^2^Department of Pharmaceutical Biology and Biotechnology, Institute of Pharmaceutical Sciences, University of Freiburg, Freiburg im Breisgau, Germany; ^3^Department of Chemistry, Queen’s University, Kingston, ON, Canada

**Keywords:** *Streptomyces scabies*, *Streptomyces turgidiscabies*, *Streptomyces stelliscabiei*, *Streptomyces europaeiscabiei*, biological control, Isatropolone, Pakistan

## Abstract

Potato is prone to many drastic diseases like potato common scab (CS). As no highly effective methods exist for managing CS, this study explored the possibility of using biological control. Ten bacterial strains were isolated from CS-infected potato tubers from four different locations of Punjab, Pakistan, and identified based on biochemical and molecular analysis. Analysis of 16s rDNA sequences amplified by PCR revealed the isolated bacterial strains to be *Streptomyces scabies, S. turgidiscabies* and *S. stelliscabiei*. Pathogenic islands were also confirmed among the isolates after identification of *txtAB, nec1*, and *tomA* genes with PCR amplification. One strain isolated from soil was antagonistic to the pathogenic *Streptomyces* spp., and determined to be *Streptomyces* A1RT on the basis of 16s rRNA sequencing. A methanolic extract of *Streptomyces* A1RT contained Isatropolone C, which was purified and structurally determined by ^1^H- and ^13^C-NMR, ^1^H/^1^H-COSY, HMQC, and HMBC techniques. *Streptomyces* A1RT also produced the plant growth hormone indole-3-acetic acid (IAA) with a titer of 26 μg ml^-1^ as confirmed by spectrophotometry and HPLC. In a greenhouse assay, disease severity index was established from 0 to 500. Average disease severity indexes were recorded as 63, 130.5, and 78 for *Streptomyces scabies, S. turgidiscabies* and *S. stelliscabiei*, respectively. When *Streptomyces* A1RT was applied in soil that contained one of these pathogenic isolates, the average disease severity indexes were significantly (*P* < 0.05) reduced to 11.1, 5.6 and 8.4, respectively. A significant increase in tuber weight and shoot development was also observed with the tubers treated with *Streptomyces* A1RT. The use of the plant growth-promoting *Streptomyces* A1RT against potato CS thus provides an alternative strategy to control the disease without affecting environmental, plants, animals and human health.

## Introduction

Common scab (CS) is a recurrent plant disease found all over the world (Loria et al., 1997). It is caused by the Gram positive, filamentous species of *Streptomyces* in the order of Actinomycetales. Two third of known antibiotics are produced by *Actinomycetes* and 80% of these antibiotics are produced by *Streptomyces* spp. (Ahmad et al., 2017; Wang et al., 2017). Under the genus *Streptomyces*, some species can be plant pathogens, such as *Streptomyces scabies*, causing CS of potatoes. The main pathogens of CS include *Streptomyces scabies, S. acidiscabies, S. turgidiscabies, S. europaeiscabiei* and other members, including *S. bottropensis, S. stelliscabiei* and *S. aureofaciens* (Lambert and Loria, 1989; Miyajima et al., 1998; Leiminger et al., 2013). These different species are grouped together because of their different prevalence, phenotypic and genotypic characters and they produce similar signs and symptoms to analogous hosts (Loria et al., 1997).

Pathogenic characters of these species are governed by the plant phytotoxin, thaxtomin. The predominant form of thaxtomin produced by *S. scabies* is thaxtomin A. The biosynthesis of thaxtomin A is prompted by the plant cellobiose and cellotriose (Johnson et al., 2007). Genotypically thaxtomin A production is encoded by the non-ribosomal peptide synthetase genes [*txtA* and *txtB* (*txtAB*)] and cytochrome P-450 type monooxygenase gene (*txtC*) (Healy et al., 2002). The *txtAB* genes are pathogenicity determinant, but other genes like *nec1* and *tomA* facilitate or enhance the pathogenicity, although they are not absolutely required (Kreuze et al., 1999; Healy et al., 2000; Flores-González et al., 2008; Barry et al., 2012).

The use of bacterial strains for CS suppression depends upon many factors including the interaction between antagonists and pathogens, environmental conditions, ecological and evolutionary dynamics of antagonistic strains (Kinkel et al., 2012). The use of higher densities of antagonistic *Streptomyces* are consistent with CS disease suppression (Wanner, 2007) due to the complementarity among antibiotics of *Streptomyces*. Environmental stress like soil pH, moisture and temperature can be varied in different fields or even within the same field, in the same or different seasons. The effectiveness of same antagonist can be varied among season to seasons in the same or even different fields (Prévost et al., 2006). Some potato cultivars are more sensitive to CS pathogens and they are difficult to control because their adaptiveness to harbor *S. scabies* is greater as compared to antagonistic strain (Neeno-Eckwall et al., 2001).

There is long-standing interest in the use of plant growth-promoting bacteria as a strategy for sustainable agriculture (Shennan, 2008). The use of natural or manipulated microbial communities which suppress phytopathogens not only has the potential to reduce disease input but also reduce the need of chemical inputs such as pesticides and fungicides (Emmert and Handelsman, 1999). In Nature, plants benefit from symbiotic bacteria that counteract pathogens and thus inhibit the growth of disease in the plant (Lorang et al., 1995). PGRP are focused for use as biological control agent due to beneficial characters which include the production of bioactive compounds (Samac et al., 2003) and secondary metabolites including antibiotics, insecticides, and pesticides (Berdy, 2005). In addition, plant growth-promoting bacteria enhance plant growth by producing phytohormones like auxins (Lee et al., 2004), siderophores (Ryan et al., 2008), and by activating plant defense pathways (Lin et al., 2012).

In the past, microorganisms have shown good potential as plant-growth promoting bacteria to enhance the growth and yield of rice (Lucas et al., 2009), wheat (Majeed et al., 2015), beans (Perez-Montano et al., 2014), maize (Qaisrani et al., 2014), and soya bean (Cassán et al., 2009). But limited data is available for growth-promotion (Abbas et al., 2014) and disease suppression (Kobayashi et al., 2015) in potatoes. *Streptomyces* spp. have been reported as plant growth promoting bacteria due to beneficial effects in resisting pathogens (Neeno-Eckwall et al., 2001), producing lytic enzymes (Prapagdee et al., 2008), resisting environmental effects (Coombs and Franco, 2003), and secreting siderophores and phytohormones (Berg, 2009; Khamna et al., 2009) which are beneficial in interacting with the plant rhizosphere (Khamna et al., 2009).

In present study, potato CS pathogens were isolated from Punjab, Pakistan. The antagonistic bacterial strains were isolated from a potato field with no CS history over the past 5 years. It was hypothesized that such fields may contain microorganisms which augment plant growth and help in disease suppression in potato crops. With this aim, a study was conducted for isolation and characterization of potato CS pathogens. Sampling was also performed to isolate antagonistic bacterial strains. We described the potential of *Streptomyces* A1RT as plant growth- promoting and CS disease suppressing in potato crop. To our best knowledge this is the first report of biocontrol against four major CS pathogens.

## Materials and Methods

### Sample Collection, Bacterial Isolation and Identification

CS-infected potato tubers were collected from Potato Research Institute (PRI), Sahiwal, Pakistan. Potato samples were shifted to the Department of Microbiology and Molecular Genetics, University of the Punjab Lahore, Pakistan. Samples were graded according to their disease severity as very low, low, moderate, moderately high, high and very high depending upon the percentage 10, 20, 40, 60, 80, and 90% of CS symptoms respectively.

Fourteen potato samples with various CS symptoms were processed. All potato samples were washed with sterile distilled water and then disinfected with 2.8% sodium hypochlorite (NaOCl) solution, followed by rinsing three times with sterile distilled water. The scabby lesions were carefully cut with sterile scalpel and homogenized with 2 mL Tris–HCl (pH 7.2) in a Eppendorf tube followed by incubation for 10 min at 50°C in incubator. The homogenized mixture was further diluted three times with Tris–HCl (pH 7.2). For each aliquot, 100 μl diluted sample was spread on yeast malt extract (YME) agar plates (Meng et al., 2011) and incubated at 28°C for 7 days. Selected single bacterial colonies were spread on another YME agar plate and physical growth characters were recorded for each *Streptomyces* spp. Ten bacterial isolates having *Streptomyces*-like growth characters were isolated and purified on additional YME agar plates. These *Streptomyces* spp. were identified based on typical growth characters, diffusible pigments and spore colors compared with already identified *Streptomyces* spp. (positive controls kindly provided by Dr. Jurgen Leiminger, Freising, Germany).

For isolation of antagonistic bacterial spp., soil samples were collected from a potato growing field that have had very low or no potato CS history over past 5 years. Antibiotic- producing bacterial spp., especially *Streptomyces* spp., were selected as potential CS antagonists (Kharel et al., 2005).

### DNA Extraction and Polymerase Chain Reaction (PCR)

For DNA extraction, the selected *Streptomyces* colonies were grown in YME broth for 5 days at 28°C. Each culture was centrifuged and the pellets were subjected to DNA extraction by using the Gene-JET genomic DNA purification kit (Thermo Scientific, United States). The extracted DNA was quantified by absorbance at 260 nm (NanoDrop, Thermo Scientific, United States). High quality DNA (A_260_/A_280_ ratio was 1.7) samples were subjected to PCR amplification by using 16S rRNA universal primers (Edwards et al., 1989) for the characterization of the *Streptomyces* spp. Each PCR reaction contained 1 μL DNA template, 2.5 μL PCR buffer (MgCl_2_), 1 μL *Taq* DNA polymerase, 1 μL 2 mM dNTPs, 1 μL 10 pmol of each primer and 18.5 μL H_2_O to afford a total volume of 25 μl. PCR amplification was performed in BioRad Thermocycler, United States. The PCR reaction was carried out with an initial denaturation at 94°C for 3 min followed by 35 cycles of denaturation at 94°C for 20 s, annealing at 60°C for 30 s, and extension at 72°C for 40 s. Following the cycling, a final extension at 72°C for 3 min was performed.

To identify *Streptomyces* strains, species-specific primers were used for *S. scabies* (Lehtonen et al., 2004), *S. turgidiscabies, S. acidiscabiei* (Tagawa et al., 2008), *S. europaeiscabiei, S. stelliscabiei* and *S. bottropensis* (Wanner, 2006). The 16S-23S internal transcribed spacer (ITS) region was amplified by using the ITS-F and ITS-R primer pair (Song et al., 2004). The amplicon was digested with *Hpy99I* (New England Biolabs) which cut the ITS site at 1629-1633 nucleotide position. The restriction pattern was then visualized by gel electrophoresis. The PCR products were sequenced by GACT, Germany. The 16S rRNA sequences have been submitted to European Nucleotide Database EMBL with the accession numbers KU560917, LN908787 and LN908789.

### Identification of a Pathogenicity Island

Polymerase chain reaction primers were designed to identify pathogenicity island (PAI) of the susceptible pathogenic *Streptomyces* strains. PCR amplification was performed by using *txtAB* gene, *tomA* gene (Wanner, 2006) and *nec1* gene (Bukhalid et al., 1998). PCR was performed as conditions described above except annealing temperature was set as 48°C, 55°C and 60°C for the *txtAB, tomA* and *nec1* genes, respectively. All the identified genes have been deposited in the NCBI with the accession numbers KX842598-606.

### Bacterial Inhibition Assay

To check the antibacterial activity of the antagonistic *Streptomyces* spp. against pathogenic CS causing *Streptomyces* strains, a disk diffusion assay was performed (CLSI, 2015). Twelve *Streptomyces* spp. were grown separately in YME broth for 5–7 days at 28°C in shaking incubator. An aliquot of 100 μL of pathogenic *Streptomyces* spp. culture was spread as a lawn on YME agar plates with the help of Rattler^TM^ plating beads (Zymo Research Cooperation, United States). After drying the plates, individual filter paper disks were used to absorb 25 μL of MeOH extract from each antagonistic *Streptomyces* isolate. The filter disks were then applied to the YME agar plates, which were then placed in incubator at 28°C for 3 days in upright position. After incubation, the inhibition zone around the filter disks were recorded in millimeters (mm). To confirm the antibacterial activity of Isatropolone C containing fractions, the bacterial inhibition assay was performed using Isatropolone C dissolved in methanol.

### Indole-3-Acetic Acid Production and Analysis by HPLC-DAD-MS

To determine the potential of antagonistic *Streptomyces* isolates as plant growth-promoting organisms, colorimetric estimation of IAA was performed as described by Gordon and Weber (1951). Bacterial strains were grown in triplicates for 5–7 days at 28°C, 180 rpm, in ISP-2 liquid medium supplemented with 500 μg mL^-1^ L-tryptophan (filter sterilized). After incubation, the culture was centrifuged at 14,000 rpm for 15 min. One milliliter of supernatant was mixed with 2 mL Salkowski’s reagent (prepared by adding 1 mL of 0.5 M FeCl_2_ to 50 mL 35% perchloric acid) and incubated in the dark at room temperature for half an hour. Development of pink color was observed as an indicator of IAA production. IAA estimation was performed by measuring the absorbance at 535 nm (Uvikon 933). A standard curve for IAA was generated and the IAA titer was expressed as μg mL^-1^.

Indole-3-acetic acid production was also determined by HPLC-DAD-MS. Culture supernatants generated as described above were acidified to pH 2.5 with 1N HCl and extracted twice with an equal volume of ethyl acetate. The organic phase was evaporated in vacuo and the resulting powder redissolved in 5 mL MeOH. HPLC analysis was performed on a Agilent 1100 system equipped with a diode array detector and a quadrupole mass detector. The crude methanolic extract of IAA was diluted 10 times and 20 μL was injected onto a reverse phase XBridge C18 column (3.5 μm, 100 mm × 4.6 mm) equilibrated with 95:5 methanol/water. The extract was resolved isocratically at a flow rate of 0.5 mL min^-1^. The retention time of IAA was identified by comparison to a standard sample (Sigma-Aldrich).

### Secondary Metabolite Production and Analysis by HPLC-DAD-MS

Antagonistic strains were cultivated in 500 mL shake flasks containing 150 mL TSB medium for 24–48 h at 28°C, 180 rpm. Ten milliliters of this preculture was used to inoculate 150 mL of YME medium and incubated for 3–5 days under the same conditions. After cultivation, the cells were removed by centrifugation and the culture supernatant was extracted twice with 100 mL ethyl acetate. After concentration *in vacuo*, the extract was dissolved in methanol and used for HPLC-DAD-MS analysis. The HPLC conditions were identical to those used for IAA analysis.

### Purification and Structural Elucidation of Isatropolone C

A 100 mL TSB pre-culture of *Streptomyces* sp. A1RT was used to inoculate 10 L YME, which was then incubated for 3 days at 28°C, 180 rpm. After incubation, the cells were removed by centrifugation and the supernatant extracted twice with an equal volume of ethyl acetate. The solvent was evaporated in vacuo to produce a powder. Acetylation was performed with acetic anhydride and pyridine. One hundred milligrams of the powdered extract was re-suspended in 1 mL pyridine in a twin neck round bottom flask with continuous stirring. The flask was flushed with nitrogen and 100 μL acetic anhydride was added by drops via syringe. The reaction was allowed to proceed for 30 min at room temperature, whereupon the reaction was concentrated in vacuo. The resulting powder was redissolved in 5 mL methanol and applied to an Oasis^®^ HLB20 35 cc cartridge (6 g). The cartridge was eluted with a step gradient of methanol, ranging from 20 to 100%. Each fraction was analyzed by HPLC-DAD-MS. Fractions containing acetylated Isatropolone C were concentrated in vacuo.

Acetylated Isatropolone C was further purified by semi-preparative HPLC (Agilent Technologies) using a Zorbax B-C18 (9.4 mm × 150 mm) pre-column and a Zorbax B-C18 (9.4 mm × 20 mm) main column. A concentrated methanol solution of crude compound was applied to the column and eluted with acetonitrile / 0.5% acetic acid as buffer A and water / 0.5% acetic acid as buffer B at a flow rate of 2 mL min^-1^. Purified acetylated Isatropolone C was dissolved in CD_3_OD and analyzed by ^1^H-NMR (400 MH_Z_) and ^13^C-NMR (100 MH_Z_) on a Bruker DRX-500 spectrometer (Bruker, Karlsruhe, Germany). 2D-NMR (^1^H/^1^H-COSY, HMQC, and HMBC) and high resolution MS were also used to confirm the structure of the compound.

### Pathogenicity Test on Potato Tubers

The pathogenicity of the isolated *Streptomyces* toward potato tubers was tested as described by Wanner (2006). *S. europaeiscabiei* strain G1 was used as a positive control (kindly provided by Dr. Jurgen Leiminger, Freising, Germany). To confirm the normal growth pattern of potato tubers, one control without any bacterial inoculation was also used. The *Streptomyces* isolates were inoculated in 100 mL YME broth in 250 ml flasks and placed in shaking incubator at 28°C, 180 rpm, for 5–7 days until they attained 10^6^ CFU mL^-1^ concentration. Each culture was centrifuged at 10,000 rpm for 2 min to collect cells. The cell pellet was re-suspended in 100 mL^-1^ sterile distilled water to prepare vermiculite. Medium size pots (8 L) were substrate-filled with autoclaved Compo Sana Universal^®^ (Munster Germany). One hundred mL vermiculite inoculum was added and spread equally in each substrate filled pot, in three replicates. Potato tubers were surface sterilized by 0.5% bleach for 10–15 min and washed with sterilized water. The potatoes were twice grown in a greenhouse, once in September 2015 and a second time in March 2016. The average temperature was 22–25°C. Plants were watered as required and continuously monitored for growth. After 3 months the progeny tubers were harvested, weighed, and the length of shoots and roots recorded. The tubers were also scored for disease. Pathogenic *Streptomyces* spp. were again isolated from infected lesions in harvested tubers to confirm the source of CS.

### Statistical Analysis

Statistical analysis was performed using SPSS software (IBM SPSS Statistics, Version 21). Data was collected in triplicates and subjected to one-way analysis of variance (ANOVA). Compared means were separated by Duncan’s multiple range test (DMRT). Values of *p* < 0.05 were considered statistically significant.

## Results

### Identification and Molecular Characterization of *Streptomyces* Species Causing CS

Out of ten *Streptomyces* spp. isolated from CS infected tubers, seven CS-causing *Streptomyces* spp. were identified based on 16S rRNA analysis by using specific primers designed for different CS species (Supplementary Table S1). Two isolates were identified as *S. scabies*, four as *S. turgidiscabies*, and one as *S. stelliscabiei*. The two *S. scabies* isolates were distinguished from *S. europaeiscabiei* by the presence of a *Hpy99I* restriction enzyme site within the 16S-23S ITS region of the 16S rRNA sequences (Flores-González et al., 2008).

**FIGURE 1:**
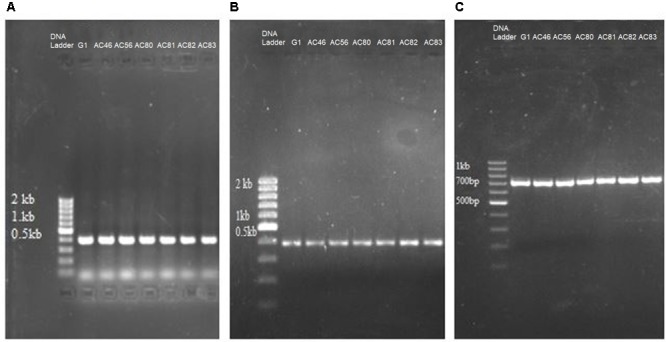
PCR detection of genes related to CS pathogenicity in seven *Streptomyces* isolates. Shown are the agarose electrophoresis gels of PCR reactions amplifying **(A)** *txtAB*; **(B)** *nec1*; **(C)** *tomA*. The name of each isolate (G1, AC46, AC56, AC80, AC81, AC82, AC83) is shown above the respective lanes of the gels.

Polymerase chain reaction was used to identify genes in the isolates that encode pathogenicity. Seven out of ten *Streptomyces* isolates, representing *S. scabies, S. turgidiscabies* and *S. stelliscabiei*, were subjected to PCR using primers to amplify *txtAB* gene. Beside *txtAB* gene, *nec1* and *tomA* genes were detected by PCR amplification (**Figures 1A–C**). Neither *nec1* nor *tomA* were detected in the *txtAB*-negative *Streptomyces* isolates but both genes were present among all *txtAB*-positive *Streptomyces* isolates. Accession numbers are summarized in Supplementary Table S2.

*Streptomyces* A1RT, which shows activity against *S. scabies* (see below), did not test positive for *txtAB, nec1* and *tomA* by PCR, indicating that this strain is non-pathogenic and hence, does not produce thaxtomin A.

### Isolation of *Streptomyces* Strains With Activity Against *S. scabies* and Isolation of Isatropolone A

**FIGURE 2:**
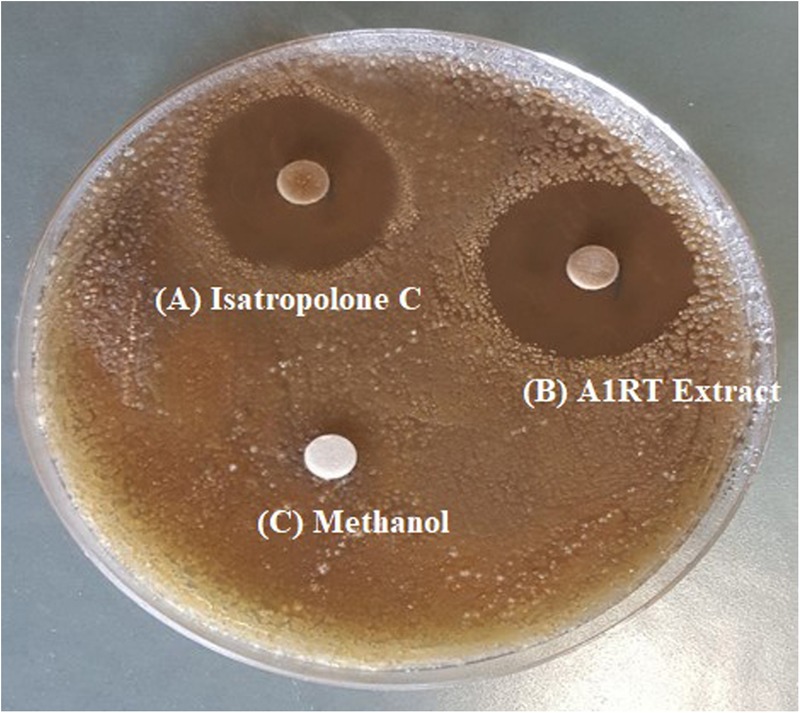
Inhibition of *S. scabies* by disk diffusion assay. *S. scabies* strain AC46 was grown on a solid medium plate in the presence of filter paper disks containing **(A)** Purified Isatropolone C dissolved in methanol. **(B)** Crude extract of *Streptomyces* A1RT **(C)** Methanol only.

Streptomyces strains were isolated from soil, showing activity against *S. scabies.* Out of twelve antagonistic *Streptomyces* strains, extracts of seven strains showed bioactivity (inhibition zone ranged from 6 to 26 mm) (Supplementary Table S3). The highest extract activity (26 mm) against *S. scabies* was derived from *Streptomyces* A1RT (Supplementary Table S3 and **Figure 2**). The *Streptomyces* A1RT extract was also active against *S. turgidiscabies* and *S. stelliscabiei* (data not shown).

**FIGURE 3:**
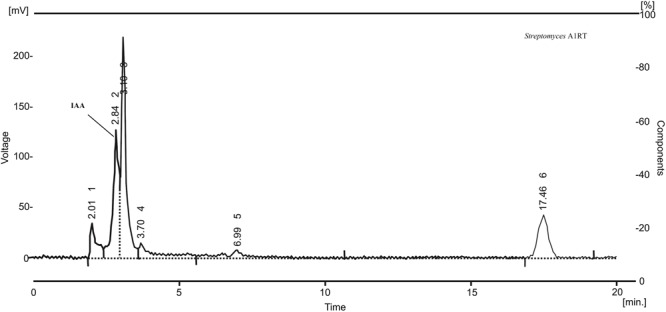
HPLC chromatogram of an extract from *Streptomyces* A1RT. The peak for indole-3-acetic acid (IAA) is shown. The retention time of the peak is identical to a standard sample of indole-3-acetic acid.

The twelve strains were also analyzed for their ability to produce IAA. The best producer, *Streptomyces* A1RT, produced IAA at a concentration of 26 μg mL^-1^ after 4 days of incubation at 28°C. Production was confirmed by HPLC analysis of the extract (**Figure 3**).

One prominent metabolite produced by *Streptomyces* A1RT was selected for purification and structural characterization. Through the targeted mass fractionation, a dark yellow to orange amorphous powder was isolated which showed antibiotic activity. The molecular formula was predicted to be C_24_H_24_O_10_ by high resolution MS (Supplementary Figure S1) based on the observed molecular ion [M-H]^-^; with *m/z* 471.1302 (calcd. for C_24_H_24_O_10_, *m/z* 471.1302), which corresponds to a hydrogen deficiency index of 13.

**FIGURE 4:**
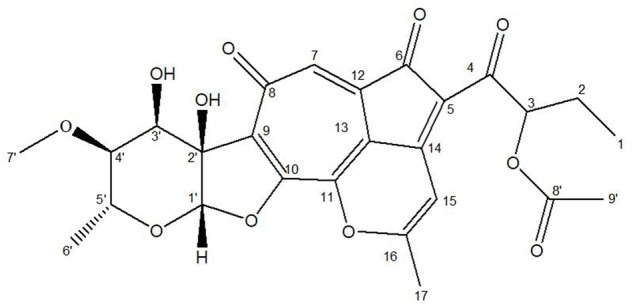
Chemical structure of acetylated Isatropolone C.

Due to the instability of this compound during purification, the crude extracts of the fermentation broth were subjected to global acetylation to protect putative reactive hydroxy groups in the molecule (Yu et al., 2012). A monoacylated molecule with *m/z* 513.2 ([M-H]^-^) was detected in the extract, which was isolated through a sequence of chromatographic steps to yield 5 mg of purified compound. Single and 2-dimensional NMR spectroscopic data (Supplementary Table S4 and Supplementary Figures S3–S7) for the compound was consistent with a *O*-acylated derivative of the recently described Isatropolone C (**Figure 4**) (Cai et al., 2017). The absorbance spectrum (Supplementary Figure S2) revealed distinct maxima at 300–350 nm and closely resembled the spectrum reported for Isatropolone A (Cai et al., 2017).

### Growth Promotion and CS Disease Suppression by *Streptomyces*

**FIGURE 5:**
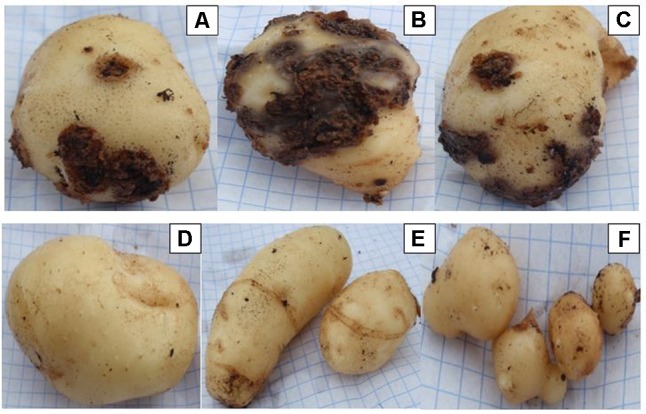
Potato tubers harvested from potting soil treated with *Streptomyces* strains **(A)** AC46; **(B)** AC56; **(C)** AC80; **(D)** A1RT+AC46; **(E)** A1RT+AC56; and **(F)** A1RT+AC80.

**FIGURE 6:**
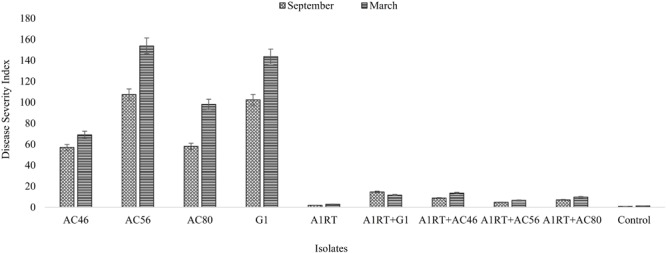
Graphical representation of disease severity index calculated after harvesting. Error bars represent standard error. The results shown in triplicates (*p* < 0.05).

Three *Streptomyces* isolates (AC46, AC56, and AC80) produced CS lesions on surface of potatoes while no observable CS symptoms were detected for tubers infested with combination of pathogens and antagonistic bacterial strain (**Figure 5**). The CS symptoms ranged from superficial to deep pitted scars and protruding CS lesions. In order to calculat disease severity, disease severity (DS) index was established ranging from 0 to 500 as described by Wanner (2009). All tested organisms having DS index higher than 20 were considered pathogenic (Wanner, 2009). The DS index of test strains were ranged from 102, 57, 107 and 58 for *S. europaeiscabiei* strain G1, AC46, AC56 and AC80, respectively. The DS index was significantly (*p* < 0.05) reduced to 14.7, 1.8, 4.7 and 1.4 for strains G1, AC46, AC56 and AC80, respectively when used in combination with *Streptomyces* A1RT (**Figure 6**). The DS index was 0 for tuber exposed to *Streptomyces* A1RT and 2.3 for non-infested tubers.

**FIGURE 7:**
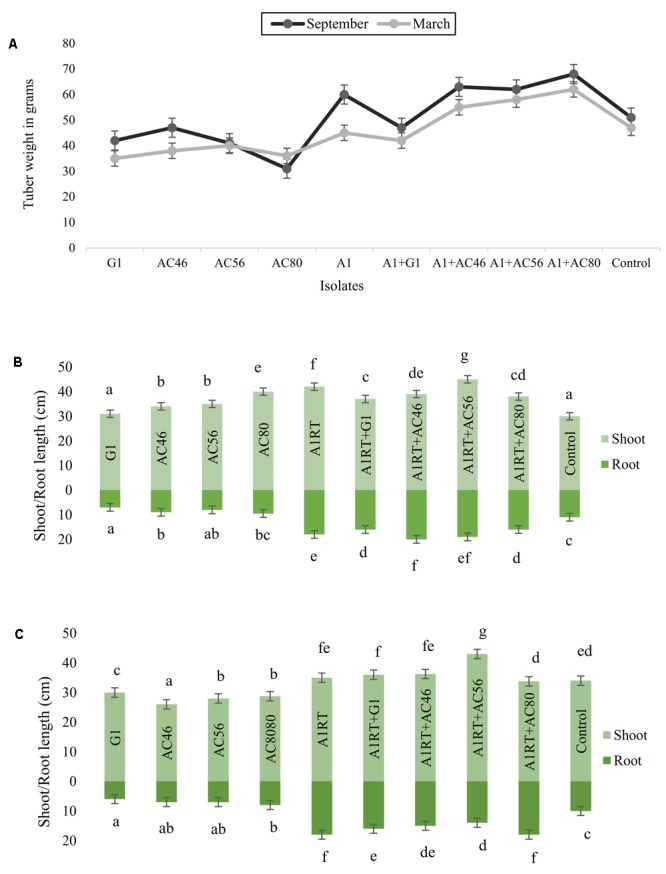
Graphical representation of plant growth promoting effects **(A)**: Increase in tuber growth was seen when using *Streptomyces* A1RT with different CS pathogens. **(B)** Shoot and root growth in September **(C)** Shoot and root growth in March. Error bars representing mean ± S.E of triplicates. The letters above the bars indicate significant differences between treatments calculated by Duncan’s multiple range test (DMRT).

Growth promotion potential was assessed in terms of relative gain in tuber weight, root and shoot development to the plants exposed to *Streptomyces* A1RT as compared to the plants exposed to CS pathogens only. For example, when tubers inoculated with CS pathogens, average tuber weight was measured as 40 g which significantly (*p* < 0.05) increased up to 60 g when used in combination with *Streptomyces* A1RT. Similarly, an average 5–10 cm increase in shoot and root length (**Figure 7**) was observed in plants inoculated with pathogens and *Streptomyces* A1RT as compared to plants exposed to CS pathogens only. However, not a significant difference (*p* > 0.05) was observed between experiments conducted in March and September.

## Discussion

The history of controlling soil borne pathogens by using *Streptomyces* spp. as biocontrol agents is more than 50 years old. In addition to nutrient competition, *Streptomyces* spp. have multiple mechanisms which can be used to control soil pathogens, including production of antibiotics, degradative enzymes, and nitrous oxide (Cohen and Mazzola, 2006; Mahmoudi et al., 2011). Among them, disease control by producing antibiotics remained an active and most promising strategy. Additionally, *Streptomyces* spp. have potential as plant growth-promoting microorganisms to enhance carbon and nitrogen resources and efficiently competing in the rhizosphere (Schlatter et al., 2008). Therefore, the ultimate benefit of using plant growth promoting bacteria is not only in disease suppression or enhancement of plant growth factors, but also development of sustainable agricultural practices. This latter factor is especially important in developing countries like Pakistan where almost 250 kg nitrogen and 150 kg each of phosphorus and potash is used per hectare for potato production (Naqqash et al., 2016). To this end, we report the isolation of the strain *Streptomyces* A1RT from field suppressive soil where CS disease symptoms were negligible over the period of 5 years.

CS disease-promoting bacteria are an evolving threat to potato crops. Although *S. scabies* is the best known causative agent of CS disease, it was reported that *S. turgidiscabies* also has the pathogenicity-related genes that encode the production of the phytotoxin thaxtomin A. Although *S. turgidiscabies* shows high sequence similarity with *S. scabies* (Kers et al., 2005), both species are different (Miyajima et al., 1998). Moreover, *S. turgidiscabies* was reported as more of a CS threat in terms of growth characters, virulence, and ability to grow even in humid environmental conditions (Hiltunen et al., 2009). Such features gave rise to the hypothesis that *S. turgidiscabies* may be evolving as an emerging plant pathogen and have gained decisive pathogenic factors from *S. scabies* (Loria et al., 2006). Previous data also suggest that due to the versatility and adaptability of *S. turgidiscabies* to different environmental conditions, it is possible that *S. turgidiscabies* will displace *S. scabies* from the environment in the future. Therefore, it is important to find antagonistic organisms that act against this emerging CS pathogen. In past studies, bacterial species such as *Bacillus* spp. (Han et al., 2005), *Pseudomonas* spp. (Arseneault et al., 2013), *S. albidoflavus* (Hayashida et al., 1989) and *S. diastatochromogenes* (Neeno-Eckwall et al., 2001) have been used as biological control of potato CS. Likewise, *Streptomyces* spp. have been used as biological control agents against *S. scabies* (Prévost et al., 2006) and *S. turgidiscabies* (Hiltunen et al., 2009). However, to our knowledge *Streptomyces* A1RT is the first example of a biological control that is active against *S. scabies* and *S. turgidiscabies*, but also the CS pathogens *S. europaeiscabiei* and *S. stelliscabiei*.

It is known that the plant growth-promoting hormone IAA plays a crucial role in germination rate and the development of roots and shoots (Fatima et al., 2009). In this study we demonstrate that *Streptomyces* A1RT has the ability to produce high levels of IAA. Accordingly, potato tubers treated with *Streptomyces* A1RT developed better in terms of roots elongation, number of shoots, and tuber weight. Therefore, *Streptomyces* A1RT is novel in its ability to not only suppress multiple CS pathogens but also promote potato tuber growth.

Greenhouse trials performed by using pathogenic CS strains indicated the CS disease was suppressed in pots when *Streptomyces* A1RT was present. This may be due to the production of an antibiotic compound by *Streptomyces* A1RT. Disk diffusion assays clearly indicated that a highly active compound (or compounds) are produced by *Streptomyces* A1RT. A prominent compound produced by *Streptomyces* A1RT was purified and spectroscopically shown to be Isatropolone C. Isatropolone C was recently discovered as a metabolite of *Streptomyces* Gö66 and was shown to have anti-parasitic activity (Cai et al., 2017). In our study, we report that an Isatropolone C containing extract shows high antibiotic activity.

In summary, a major goal of 21st century is to develop an environmentally friendly and sustainable agriculture. This study has identified the novel strain *Streptomyces* A1RT as a promising and effective method for biological control of multiple CS pathogens, while simultaneously promoting plant growth.

## Author Contributions

AS conducted all experimental work. ZL assisted with data anlaysis. SZ and JZ performed chromatographic steps and NMR analysis. DZ and AB assisted with experimental design and writing the manuscript.

## Conflict of Interest Statement

The authors declare that the research was conducted in the absence of any commercial or financial relationships that could be construed as a potential conflict of interest.

## References

[B1] Abbas M. T. Hamza M. A. Youssef H. H. Youssef G. H. Fayez M. Monib M. (2014). Bio-preparates support the productivity of potato plants grown under desert farming conditions of north Sinai: five years of field trials. *J. Adv. Res.* 5 41–48. 10.1016/j.jare.2012.11.004 25685470 PMC4294711

[B2] Ahmad M. S. El-Gendy A. O. Ahmed R. R. Hassan H. M. El-Kabbany H. M. Merdash A. G. (2017). Exploring the antimicrobial and antitumor potentials of *Streptomyces* sp. AGM12-1 Isolated from Egyptian Soil. *Front. Microbiol.* 8:438. 10.3389/fmicb.2017.00438 28348553 PMC5346535

[B3] Arseneault T. Goyer C. Filion M. (2013). Phenazine production by *Pseudomonas* sp. LBUM223 contributes to the biological control of potato common scab. *Phytopathology* 103 995–1000. 10.1094/phyto-01-13-0022-r 23883153

[B4] Barry S. M. Kers J. A. Johnson E. G. Song L. Aston P. R. Patel B. (2012). Cytochrome P450-catalyzed L-tryptophan nitration in thaxtomin phytotoxin biosynthesis. *Nat. Chem. Biol.* 8 814–816. 10.1038/nchembio.1048 22941045 PMC3522571

[B5] Berdy J. (2005). Bioactive microbial metabolites. *J. Antibiot.* 58 1–26. 10.1038/ja.2005.1 15813176

[B6] Berg G. (2009). Plant–microbe interactions promoting plant growth and health: perspectives for controlled use of microorganisms in agriculture. *Appl. Microbiol. Biotechnol.* 84 11–18. 10.1007/s00253-009-2092-7 19568745

[B7] Bukhalid R. A. Chung S. Y. Loria R. (1998). *nec1*, a gene conferring a necrogenic phenotype, is conserved in plant-pathogenic *Streptomyces* spp. and linked to a transposase pseudogene. *Mol. Plant Microbe Interact.* 11 960–967. 10.1094/mpmi.1998.11.10.960 9768513

[B8] Cai X. Shi Y. M. Pohlmann N. Revermann O. Bahner I. Pidot S. J. (2017). Structure and biosynthesis of isatropolones, bioactive amine-scavenging fluorescent natural products from *Streptomyces* Go66. *Angew. Chem. Int. Ed. Engl.* 56 4945–4949. 10.1002/anie.201701223 28371116

[B9] Cassán F. Perrig D. Sgroy V. Masciarelli O. Penna C. Luna V. (2009). *Azospirillum brasilense* Az39 and *Bradyrhizobium japonicum* E109, inoculated singly or in combination, promote seed germination and early seedling growth in corn (*Zea mays* L.) and soybean (*Glycine max* L.). *Eur. J. Soil Biol.* 45 28–35. 10.1016/j.ejsobi.2008.08.005

[B10] Cohen M. F. Mazzola M. (2006). Resident bacteria, nitric oxide emission and particle size modulate the effect of *Brassica napus* seed meal on disease incited by *Rhizoctonia solani* and *Pythium* spp. *Plant Soil* 286 75–86. 10.1007/s11104-006-9027-1

[B11] Coombs J. T. Franco C. M. (2003). Isolation and identification of actinobacteria from surface-sterilized wheat roots. *Appl. Environ. Microbiol.* 69 5603–5608. 10.1128/AEM.69.9.5603-5608.2003 12957950 PMC194995

[B12] Edwards U. Rogall T. Blocker H. Emde M. Bottger E. C. (1989). Isolation and direct complete nucleotide determination of entire genes. Characterization of a gene coding for 16S ribosomal RNA. *Nucleic Acids Res.* 17 7843–7853. 10.1093/nar/17.19.7843 2798131 PMC334891

[B13] Emmert E. A. Handelsman J. (1999). Biocontrol of plant disease: a (Gram-) positive perspective. *FEMS Microbiol. Lett.* 171 1–9. 10.1111/j.1574-6968.1999.tb13405.x 9987836

[B14] Fatima Z. Saleemi M. Zia M. Sultan T. Aslam M. Rehman R. (2009). Antifungal activity of plant growth-promoting rhizobacteria isolates against *Rhizoctonia solani* in wheat. *Afr. J. Biotechnol.* 8 219–225. 10.1111/1751-7915.12158 25219642 PMC4408174

[B15] Flores-González R. Velasco I. Montes F. (2008). Detection and characterization of *Streptomyces* causing potato common scab in Western Europe. *Plant Pathol.* 57 162–169. 10.1111/j.1365-3059.2007.01734.x

[B16] Gordon S. A. Weber R. P. (1951). Colorimetric estimation of indoleacetic acid. *Plant Physiol.* 26 192–195. 10.1104/pp.26.1.192 16654351 PMC437633

[B17] Han J. S. Cheng J. H. Yoon T. M. Song J. Rajkarnikar A. Kim W. G. (2005). Biological control agent of common scab disease by antagonistic strain *Bacillus* sp. sunhua. *J. Appl. Microbiol.* 99 213–221. 10.1111/j.1365-2672.2005.02614.x 15960681

[B18] Hayashida S. Choi M.-Y. Nanri N. Yokoyama M. Uematsu T. (1989). Control of potato common scab with an antibiotic biofertilizer produced from swine feces containing *Streptomyces albidoflavus* CH-33. *Agric. Biol. Chem.* 53 349–354. 10.1080/00021369.1989.10869326

[B19] Healy F. G. Krasnoff S. B. Wach M. Gibson D. M. Loria R. (2002). Involvement of a cytochrome P450 monooxygenase in thaxtomin a biosynthesis by *Streptomyces acidiscabies*. *J. Bacteriol.* 184 2019–2029. 10.1128/jb.184.7.2019-2029.2002 11889110 PMC134914

[B20] Healy F. G. Wach M. Krasnoff S. B. Gibson D. M. Loria R. (2000). The *txtAB* genes of the plant pathogen *Streptomyces acidiscabies* encode a peptide synthetase required for phytotoxin thaxtomin A production and pathogenicity. *Mol. Microbiol.* 38 794–804. 10.1046/j.1365-2958.2000.02170.x 11115114

[B21] Hiltunen L. H. Ojanperä T. Kortemaa H. Richter E. Lehtonen M. J. Valkonen J. P. T. (2009). Interactions and biocontrol of pathogenic *Streptomyces* strains co-occurring in potato scab lesions. *J. Appl. Microbiol.* 106 199–212. 10.1111/j.1365-2672.2008.03992.x 19054229

[B22] Johnson E. G. Joshi M. V. Gibson D. M. Loria R. (2007). Cello-oligosaccharides released from host plants induce pathogenicity in scab-causing *Streptomyces* species. *Physiol. Mol. Plant Pathol.* 71 18–25. 10.1016/j.pmpp.2007.09.003

[B23] Kers J. A. Cameron K. D. Joshi M. V. Bukhalid R. A. Morello J. E. Wach M. J. (2005). A large, mobile pathogenicity island confers plant pathogenicity on *Streptomyces* species. *Mol. Microbiol.* 55 1025–1033. 10.1111/j.1365-2958.2004.04461.x 15686551

[B24] Khamna S. Yokota A. Lumyong S. (2009). Actinomycetes isolated from medicinal plant rhizosphere soils: diversity and screening of antifungal compounds, indole-3-acetic acid and siderophore production. *World J. Microbiol. Biotechnol.* 25 649–655. 10.1007/s11274-008-9933-x

[B25] Kharel M. K. Shepherd M. D. Nybo S. E. Smith M. L. Bosserman M. A. Rohr J. (2005). Isolation of *Streptomyces* species from soil. *Curr. Protoc. Microbiol.* Chapter 10:Unit 10E.4. 10.1002/9780471729259.mc10e04s19 21053254

[B26] Kinkel L. L. Schlatter D. C. Bakker M. G. Arenz B. E. (2012). Streptomyces competition and co-evolution in relation to plant disease suppression. *Res. Microbiol.* 163 490–499. 10.1016/j.resmic.2012.07.005 22922402

[B27] Kobayashi Y. O. Kobayashi A. Maeda M. Someya N. Takenaka S. (2015). Biological control of potato scab and antibiosis by antagonistic *Streptomyces* sp. WoRs-501. *J. Gen. Plant Pathol.* 81 439–448. 10.1007/s10327-015-0614-y

[B28] Kreuze J. F. Suomalainen S. Paulin L. Valkonen J. P. (1999). Phylogenetic analysis of 16S rRNA genes and PCR analysis of the nec1 Gene from *Streptomyces* spp. Causing Common Scab, Pitted Scab, and Netted Scab in Finland. *Phytopathology* 89 462–469. 10.1094/phyto.1999.89.6.462 18944717

[B29] Lambert D. H. Loria R. (1989). *Streptomyces scabies* sp. nov., nom. rev.^†^ *Int. J. Syst. Evol. Microbiol.* 39 387–392. 10.1099/00207713-39-4-387

[B30] Lee S. Flores-Encarnación M. Contreras-Zentella M. Garcia-Flores L. Escamilla J. E. Kennedy C. (2004). Indole-3-acetic acid biosynthesis is deficient in *Gluconacetobacter diazotrophicus* strains with mutations in cytochrome c biogenesis genes. *J. Bacteriol.* 186 5384–5391. 10.1128/JB.186.16.5384-5391.2004 15292139 PMC490937

[B31] Lehtonen M. J. Rantala H. Kreuze J. F. Bång H. Kuisma L. Koski P. (2004). Occurrence and survival of potato scab pathogens (*Streptomyces* species) on tuber lesions: quick diagnosis based on a PCR-based assay. *Plant Pathol.* 53 280–287. 10.1111/j.0032-0862.2004.01009.x

[B32] Leiminger J. Frank M. Wenk C. Poschenrieder G. Kellermann A. Schwarzfischer A. (2013). Distribution and characterization of *Streptomyces* species causing potato common scab in Germany. *Plant Pathol.* 62 611–623. 10.1111/j.1365-3059.2012.02659.x

[B33] Lin L. Ge H. M. Yan T. Qin Y. H. Tan R. X. (2012). Thaxtomin A-deficient endophytic *Streptomyces* sp. enhances plant disease resistance to pathogenic *Streptomyces scabies*. *Planta* 236 1849–1861. 10.1007/s00425-012-1741-8 22922880

[B34] Lorang J. Liu D. Anderson N. Schottel J. (1995). Identification of potato scab inducing and suppressive species of *Streptomyces*. *Phytopathology* 85 261–268. 10.1094/Phyto-85-261

[B35] Loria R. Bukhalid R. A. Fry B. A. King R. R. (1997). Plant pathogenicity in the genus *Streptomyces*. *Plant Dis.* 81 836–846. 10.1094/PDIS.1997.81.8.836 30866367

[B36] Loria R. Kers J. Joshi M. (2006). Evolution of plant pathogenicity in *Streptomyces*. *Annu. Rev. Phytopathol* 44 469–487. 10.1146/annurev.phyto.44.032905.091147 16719719

[B37] Lucas J. Ramos B. Montes F. Ojeda J. Megías M. Gutierrez Mañero F. J. (2009). Use of two PGPR strains in the integrated management of blast disease in rice (*Oryza sativa*) in Southern Spain. *Field Crops Res.* 114 404–410. 10.1016/j.fcr.2009.09.013

[B38] Mahmoudi E. Tabatabaei B. E. S. Venturi V. (2011). Virulence attenuation of *Pectobacterium carotovorum* using N-Acyl-homoserine lactone degrading bacteria isolated from potato rhizosphere. *Plant Pathol. J.* 27 242–248. 10.5423/PPJ.2011.27.3.242

[B39] Majeed A. Abbasi M. K. Hameed S. Imran A. Rahim N. (2015). Isolation and characterization of plant growth-promoting rhizobacteria from wheat rhizosphere and their effect on plant growth promotion. *Front. Microbiol.* 6:198. 10.3389/fmicb.2015.00198 25852661 PMC4362341

[B40] Meng Q. Yin J. Rosenzweig N. Douches D. Hao J. J. (2011). Culture-based assessment of microbial communities in soil suppressive to potato common scab. *Plant Dis.* 96 712–717. 10.1094/PDIS-05-11-0441 30727529

[B41] Miyajima K. Tanaka F. Takeuchi T. Kuninaga S. (1998). *Streptomyces turgidiscabies* sp. nov. *Int. J. Syst. Evol. Microbiol.* 48 495–502. 10.1099/00207713-48-2-495 9731290

[B42] Naqqash T. Hameed S. Imran A. Hanif M. K. Majeed A. van Elsas J. D. (2016). Differential response of potato toward inoculation with taxonomically diverse plant growth promoting rhizobacteria. *Front. Plant Sci.* 7:144. 10.3389/fpls.2016.00144 26925072 PMC4756182

[B43] Neeno-Eckwall E. C. Kinkel L. L. Schottel J. L. (2001). Competition and antibiosis in the biological control of potato scab. *Can. J. Microbiol.* 47 332–340. 10.1139/w01-010 11358173

[B44] Perez-Montano F. Alias-Villegas C. Bellogin R. A. del Cerro P. Espuny M. R. Jimenez-Guerrero I. (2014). Plant growth promotion in cereal and leguminous agricultural important plants: from microorganism capacities to crop production. *Microbiol. Res.* 169 325–336. 10.1016/j.micres.2013.09.011 24144612

[B45] Prapagdee B. Kuekulvong C. Mongkolsuk S. (2008). Antifungal potential of extracellular metabolites produced by *Streptomyces hygroscopicus* against phytopathogenic fungi. *Int. J. Biol. Sci.* 4 330–337. 10.7150/ijbs.4.330 18825279 PMC2556053

[B46] Prévost K. Couture G. Shipley B. Brzezinski R. Beaulieu C. (2006). Effect of chitosan and a biocontrol streptomycete on field and potato tuber bacterial communities. *BioControl* 51 533–546. 10.1007/s10526-005-4240-z

[B47] Qaisrani M. M. Mirza M. S. Zaheer A. Malik K. A. (2014). Isolation and identification by 16s rRNA sequence analysis of achromobacter, azospirillum and rhodococcus strains from the rhizosphere of maize and screening for the beneficial effect on plant growth. *Pak. J. Agric. Sci.* 51 91–99.

[B48] Ryan R. P. Germaine K. Franks A. Ryan D. J. Dowling D. N. (2008). Bacterial endophytes: recent developments and applications. *FEMS Microbiol. Lett.* 278 1–9. 10.1111/j.1574-6968.2007.00918.x 18034833

[B49] Samac D. A. Willert A. M. McBride M. J. Kinkel L. L. (2003). Effects of antibiotic-producing Streptomyces on nodulation and leaf spot in alfalfa. *Appl. Soil Ecol.* 22 55–66. 10.1016/S0929-1393(02)00109-9

[B50] Schlatter D. Fubuh A. Xiao K. Hernandez D. Hobbie S. Kinkel L. (2008). Resource amendments influence density and competitive phenotypes of streptomyces in soil. *Microb. Ecol.* 57 413–420. 10.1007/s00248-008-9433-4 18704556

[B51] Shennan C. (2008). Biotic interactions, ecological knowledge and agriculture. *Philos. Trans. R. Soc. Lond. B Biol. Sci.* 363 717–739. 10.1098/rstb.2007.2180 17761466 PMC2610106

[B52] Song J. Lee S.-C. Kang J.-W. Baek H.-J. Suh J.-W. (2004). Phylogenetic analysis of *Streptomyces* spp. isolated from potato scab lesions in Korea on the basis of 16S rRNA gene and 16S–23S rDNA internally transcribed spacer sequences. *Int. J. Syst. Evol. Microbiol.* 54 203–209. 10.1099/ijs.0.02624-0 14742481

[B53] Tagawa M. Tamaki H. Manome A. Koyama O. Kamagata Y. (2008). Development of a genotyping method for potato scab pathogens based on multiplex PCR. *Biosci. Biotechnol. Biochem.* 72 2324–2334. 10.1271/bbb.80234 18776692

[B54] Wang D. Wang C. Gui P. Liu H. Khalaf S. M. H. Elsayed E. A. (2017). Identification, bioactivity, and productivity of actinomycins from the marine-derived *Streptomyces heliomycini*. *Front. Microbiol.* 8:1147. 10.3389/fmicb.2017.01147 28702007 PMC5487404

[B55] Wanner L. A. (2006). A survey of genetic variation in streptomyces isolates causing potato common scab in the United States. *Phytopathology* 96 1363–1371. 10.1094/phyto-96-1363 18943669

[B56] Wanner L. A. (2007). A new strain of *Streptomyces* causing common scab in potato. *Plant Dis.* 91 352–359. 10.1094/pdis-91-4-0352 30781174

[B57] Wanner L. A. (2009). A patchwork of *Streptomyces* species isolated from potato common scab lesions in North America. *Am. J. Potato Res.* 86 247–264. 10.1007/s12230-009-9078-y

[B58] CLSI (2015). *Clinical and Laboratory Standards Institute: Performance Standards for Antimicrobial Disk Susceptibility Tests* 12th Edn. Wayne, PA: CLSI.

[B59] Yu Z. Vodanovic-Jankovic S. Kron M. Shen B. (2012). New WS9326A congeners from *Streptomyces* sp. 9078 inhibiting *Brugia malayi* asparaginyl-tRNA synthetase. *Org. Lett.* 14 4946–4949. 10.1021/ol302298k 22967068 PMC3460372

