# Supplementary material for: Biological Control of Potato Common Scab With Rare Isatropolone C Compound Produced by Plant Growth Promoting Streptomyces A1RT
Source: Front Microbiol. 2018 May 30;9:1126. doi: 10.3389/fmicb.2018.01126 (PMC5989138; doi:10.3389/fmicb.2018.01126)
Supplement: Supplementary file 1 [file Data_Sheet_1.DOCX]

**Supporting Information:**

**Table of contents:**

**List of tables**

**Table S1:** *Streptomyces* species isolated from common scab infected tubers.

**Table S2:** Accession numbers of the pathogenicity island (PAI) related genes.

**Table S3:** Disk diffusion activity assay for extracts from isolated antagonistic *Streptomyces* spp.

**Table S4:** NMR spectroscopic data for monoacylated Isatropolone C

**List of Figures**

**Figure S1:** High resolution ESI-MS(-) spectrum of Isatropolone C.

**Figure** **S2:** Absorbance spectrum of acetylated derivative Isatroplone C.

**Figure S3:** ^1^H-NMR Spectrum of monoacylated Isatropolone C (CD_3_OD, 400 MHz)

**Figure S4:** ^13^C-NMR Spectrum of monoacylated Isatropolone C (CD_3_OD, 100 MHz)

**Figure S5:** HSQC Spectrum of monoacylated Isatropolone C

**Figure S6:** ^1^H/^1^H-COSY Spectrum of monoacylated Isatropolone C

**Figure S7:** ^1^H-^13^C-HMBC Spectrum of monoacylated Isatropolone C

**TABLE S1: *Streptomyces* species isolated from common scab infected tubers.**

| Isolates | *txtAB* | *nec1* | *tomA* |
| --- | --- | --- | --- |
| AC-42 | +ve | +ve | +ve |
| AC-46 | +ve | +ve | +ve |
| AC-55 | -ve | -ve | -ve |
| AC-56 | +ve | +ve | +ve |
| AC-57 | -ve | -ve | -ve |
| AC-80 | +ve | +ve | +ve |
| AC-81 | +ve | +ve | +ve |
| AC-82 | +ve | +ve | +ve |
| AC-83 | +ve | +ve | +ve |
| AC-86 | -ve | -ve | -ve |

**TABLE S2: Accession numbers of the pathogenicity island (PAI) related genes.**

| Isolates Name | Accession Numbers |
| --- | --- |
| *Streptomyces scabies* Strain AC-46 *txtAB* gene | KX842598 |
| *Streptomyces turgidiscabies* Strain AC-56 *txtAB* gene | KX842599 |
| *Streptomyces stelliscabiei* Strain AC-80 *txtAB* gene | KX842600 |
| *Streptomyces scabies* Strain AC-46 *nec1* gene | KX842601 |
| *Streptomyces turgidiscabies* Strain AC-56 *nec1* gene | KX842602 |
| *Streptomyces stelliscabiei* Strain AC-80 *nec1* gene | KX842603 |
| *Streptomyces scabies* Strain AC-46 *tomA* gene | KX842604 |
| *Streptomyces turgidiscabies* Strain AC-56 *tomA* gene | KX842605 |
| *Streptomyces stelliscabiei* Strain AC-80 *tomA* gene | KX842606 |

**Table S3: Disk diffusion activity assay for extracts from isolated antagonistic *Streptomyces* spp.**

| Isolate | Zone of inhibition (mm) against *Streptomyces scabies* |
| --- | --- |
| A1RT | 26 |
| A-1 | 8 |
| A-2 | 12 |
| A-3 | 6 |
| A-4 | 6 |
| A-5 | 14 |
| AC12AB | 18 |
| 14C | 10 |

**TABLE S4: NMR spectroscopic data for monoacylated Isatropolone C**

| No | ^1^H (δ, mult., *J*)^[a]^ | ^13^C^[b]^ | ^1^H-^13^C HMBC |
| --- | --- | --- | --- |
| 1 | 1.04 (t, 7.3) | 10.4, CH_3_ | 79.7, 25.1 |
| 2 | 1.89, m;  1.67, m | 25.1, CH_2_ | 194.3, 79.7, 10.4 |
| 3 | 5.85 (dd, 8.3, 3.2) | 79.7, CH | 194.3, 172.5, 25.1, 10.4 |
| 4 | - | 194.3, C |  |
| 5 | - | 114.6, C |  |
| 6 | - | 188.9, C |  |
| 7 | 7.24, s | 135.1, CH | 188.9, 185.1, 132.5, 124.4, 82.7 |
| 8 | - | 185.1, C |  |
| 9 | - | 132.5, C |  |
| 10 | - | 156.4, C |  |
| 11 | - | 145.5, C |  |
| 12 | - | 136.1, C |  |
| 13 | - | 124.4, C |  |
| 14 | - | 163.4, C |  |
| 15 | 7.74, s | 108.0, CH | 171.1, 124.4, 21.1 |
| 16 | - | 171.1, C |  |
| 17 | 2.56, s | 21.1, CH | 171.1, 108 |
| 1’ | 5.71, s | 110.6, CH | 156.4, 82.7, 68.0 |
| 2’ | - | 82.7, C |  |
| 3’ | 4.37 (d, 3.1) | 68.9, CH | 132.5, 110.6, 68.0, 82.7, 80.7 |
| 4’ | 3.31 (dd, 8.0, 3.1) | 80.7, CH |  |
| 5’ | 4.13, m | 68.0, CH | 110.6, 80.7 |
| 6’ | 1.32, (d, 6.3) | 18.4, CH_3_ | 80.7, 68.0 |
| 7’ | 3.39, s | 57.6, CH_3_ | 80.7 |
| 8’ | - | 172.5 |  |
| 9’ | 2.11, s | 20.5, CH_3_ | 172.5, 79.7 |
| [a] Recorded at 400 MHz in CD_3_OD. [b] Recorded at 100 MHz in CD_3_OD. |  |  |  |

**Figure S1**

**Figure S1:** High resolution ESI-MS(-) spectrum of Isatropolone C.

**Figure S2**

**Figure** **S2:** Absorbance spectrum of acetylated derivative Isatroplone C.

**Figure S3**


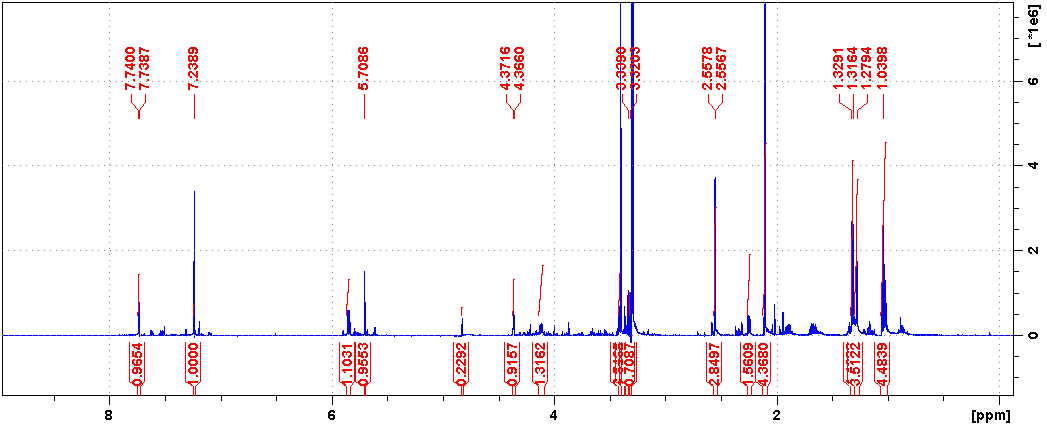


**Figure S3:** ^1^H-NMR Spectrum of monoacylated Isatropolone C (CD_3_OD, 400 MHz)

**Figure S4**


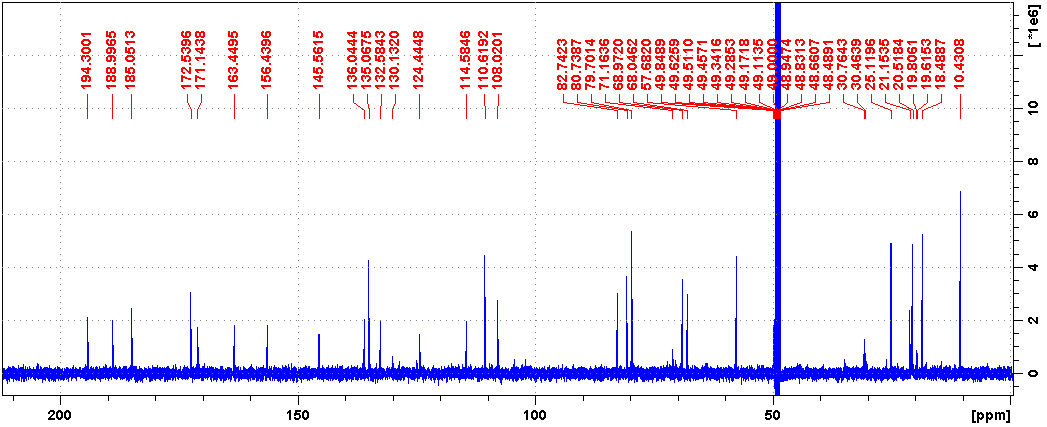


**Figure S4:** ^13^C-NMR Spectrum of monoacylated Isatropolone C (CD_3_OD, 100 MHz)

**Figure S5**


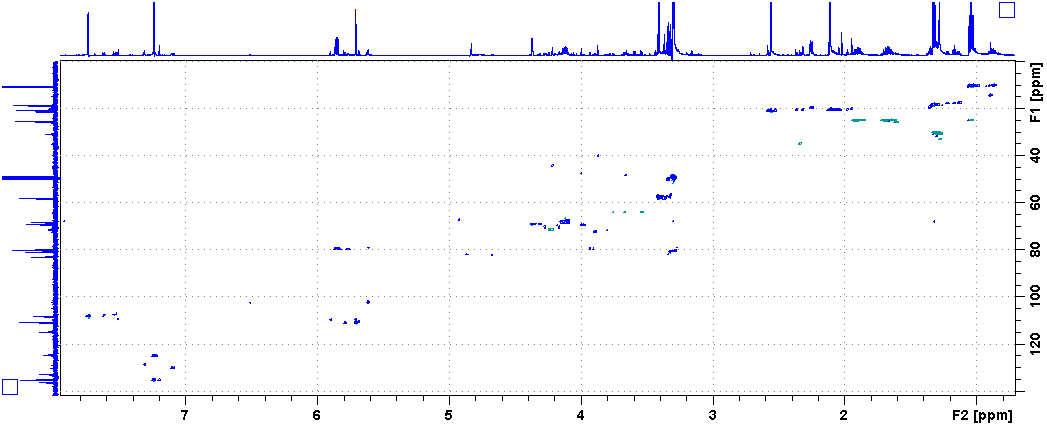


**Figure S5:** HSQC Spectrum of monoacylated Isatropolone C

**Figure S6**


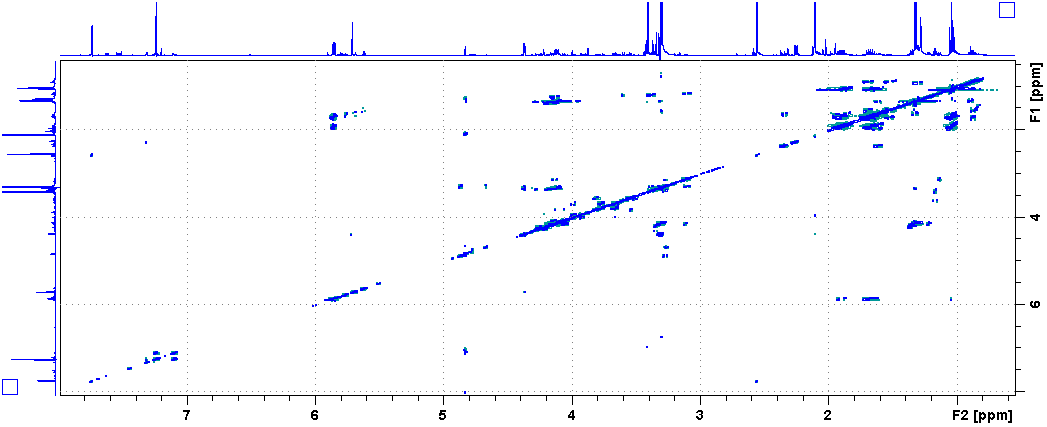


**Figure S6:** ^1^H/^1^H-COSY Spectrum of monoacylated Isatropolone C

**Figure S7**


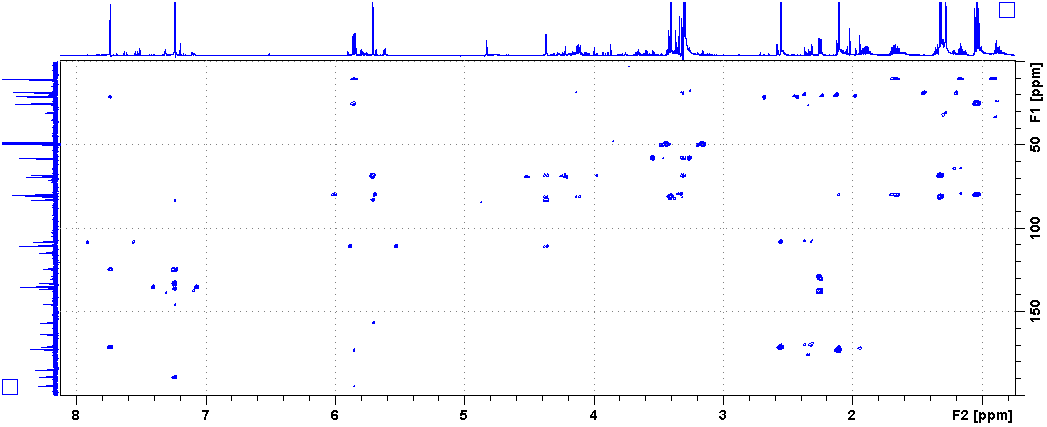


**Figure S7:** ^1^H-^13^C-HMBC Spectrum of monoacylated Isatropolone C.
